# General Practitioners involvement in enteral tube feeding at home: a qualitative study

**DOI:** 10.1186/1471-2296-8-29

**Published:** 2007-05-15

**Authors:** Sharon M Madigan, Paul Fleming, Siobhan McCann, Marion E Wright, Domhnall MacAuley

**Affiliations:** 1 Community Nutrition and Dietetics Service, Belfast Health and Social Care Trust, 67 Broadway, Belfast, Co. Antrim, BT12 6HF, UK; 2 Associate Dean, Faculty of Life and Health Sciences, University of Ulster, Shore Road, Jordanstown, Co. Antrim, BT37 0QB, UK; 3 School of Psychology, University of Ulster, Magee College, Northland Road Londonderry, UK; 4 School of Nursing Research, University of Ulster, Cromore Road, Coleraine, BT52 1SA, UK

## Abstract

**Background:**

Complex medical treatment is moving from hospital to primary care and General Practitioners (GPs) are increasingly asked to undertake new roles. There are now an estimated 19,500 patients being fed in the UK in the community on enteral tube feeding using a variety of different feeding tubes (Percutaneous endoscopic gastrostomy (PEG), Jejunostomy, or nasogastric (NG). The majority of patients are over the age of 65 years when they had artificial feeding initiated and mainly because of dysphagia. The aim of this study was to explore GPs knowledge, attitudes and skills relating to enteral feeding in the community.

**Methods:**

Semi-structured one-to-one interviews with a convenience sample of GPs in Northern Ireland.

**Results:**

Twenty-three GPs in three health boards in Northern Ireland participated in the study. Most found dealing with enteral feeding to be a predominantly negative experience. They had little involvement in patient selection for the procedure and poor or no discharge information. GPs felt inadequately trained, there was poor communication between primary and secondary care and little support. There was anger and frustration among GPs about lack of resources (funding and training), and the perception that primary care was used as a dumping ground.

**Conclusion:**

Moving complex medical treatment from secondary to primary care has major implications for GPs who should be included in the patient selection process, have adequate discharge information about their patients, be adequately resourced and have appropriate support and training.

## Background

GPs are increasingly asked to undertake roles and perform tasks that were previously undertaken in hospital. There has been a 20% increase year-on-year over the last decade in patients registered to receive Home Enteral Tube Feeding (HETF) in the UK and these patients are maintained on this regime for longer [1]. At the end of 2002 there were 19,500 adult patients in the UK on HETF, an average of about one patient per general practice and it is estimated that there are about 200,000 placed annually in the United States [2].

Moving from hospital to home releases hospital resources but has major implications for primary care and creates challenges for patients, carers and health professionals. A recent study found that most GPs (91%) had received no education regarding PEG's and 53% of the GPs surveyed had encountered problems [3]. Similarly, patients and their carers feel vulnerable and McNamara et al. [4] reported that only 19% of patients/carers felt confident in their GPs knowledge of the process. Keeping updated in treatment areas where patient numbers will only ever be small will be difficult for GPs as workloads have increased and demands of their time and resources in primary care are ever increasing [5-7].

But, questionnaire studies tell us little about the perceptions, feelings, knowledge, views, attitudes and potential barriers. In this study we interviewed a group of GPs in Northern Ireland about HETF with the aim being to explore how they feel about HETF. The specific objectives were to map barriers and training requirements in order to improve management of HETF.

## Methods

After obtaining ethical approval from the Research Ethics Committee of the University of Ulster, we wrote to all GPs registered in the Northern, Southern and Western Board Health and Social Services Board areas in Northern Ireland and invited all with past or present experience of looking after a patient who was fed via an enteral tube in the community to participate.

Thirty-one GPs responded by telephone or e-mail. Six GPs were not interviewed because a suitable interview time could not be arranged leaving a total self selected sample of 25. No financial remuneration was offered for participant's time. They were interviewed once over eight weeks during August to October 2002 at their place of work (surgeries or health centres) by the lead investigator; interviews lasted on average 30 mins. Unfortunately 2 audio-taped interviews were unclear, could not be transcribed and are not included in the analysis.

### Interviews

**Figure 1:**
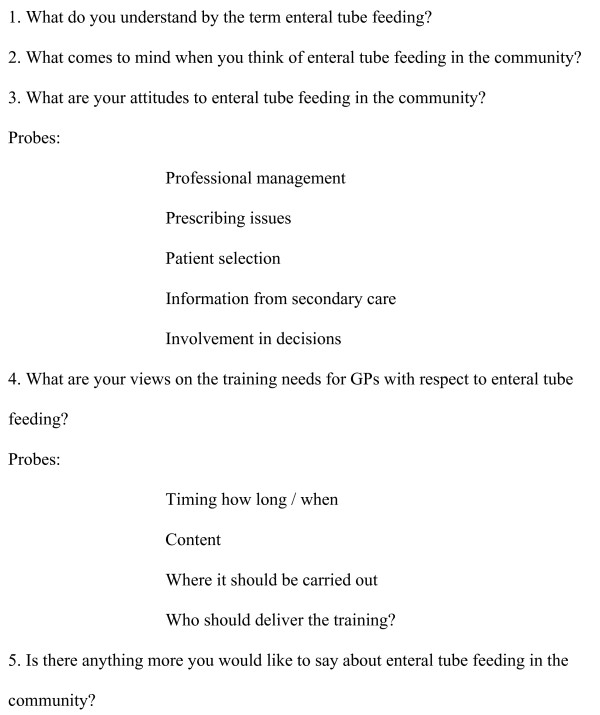
Interview Schedule with GPs.

We designed a semi structured interview based on key areas identified from discussion with primary and secondary care health professionals involved in enteral tube feeding (three community dietitians, one hospital Dietitian, one nutrition nurse, one GP, two qualitative researchers and one nurse-lecturer (Figure 1). The schedule was field tested with three GPs and two nurses and was designed to address previous experience of enteral feeding, attitudes to it, training needs and problems areas. Each area was introduced using an opening question with open ended probes used to facilitate an in-depth exploration of the area. All interviews were audio recorded.

### Analysis

All the interviews were transcribed verbatim and the transcripts checked against the original recordings. To examine analyst effects two experienced qualitative researchers (PF, SMcC) undertook an independent analysis of two randomly selected transcripts. There was agreement that the themes from this independent analysis were similar to those identified originally. The software package NUD*IST4 [8] was used for data extraction and systematic content analysis [9,10]. The interview transcripts were entered into the database and text units were allocated codes. A hierarchy of subordinate and super ordinate themes was developed. After the final transcript had been coded the transcripts were re read to search for evidence that contradicted the analysis and to ensure that the final set of themes represented the data accurately.

## Results

The study included interviews with 23 GPs. They were not chosen to be representative in age and sex of all general practitioners and participated because of their shared experience of enteral feeding. The demographic information relating to the GPs is presented in Table 1. All of the GPs had managed patients with enteral feeding tubes either in the past or were currently doing so.

**Table 1:** Demographic Information of participants

	General Practitioners
Number of participants	23
Sex	
Male	20
Female	3
Average Years since qualification	20.5
Average time working in primary care	15.6

### Previous experiences of enteral feeding

Most mentioned that their main experiences of tube feeding were when they were working in the hospital environment, which, for many, was some years previously. Several comments illustrate their lack of recent experience:

*"...enteral tube feeding was mostly when I was in hospital all those many years ago. PEG feeding well, I have had a couple of patients with it but I wouldn't say I knew an awful lot about it" *(GP 16).

*"Well... in the past obviously very little (experience) because it is a fairly new phenomenon and when I was training it was practically unheard of. But presently, or at least in the last number of years, we have had several patients in the practice, so my experience has been based on caring or at least the difficulties of caring for them" *(GP 4).

### Attitude to enteral feeding

Just under half the sample perceived HETF as a positive treatment for patients:

*"(HETF IS) positive obviously as it means that people are getting nutrition where they can't ordinarily get it" *(GP 6).

Others had serious concerns about the management of patients in the primary care setting. One GP remarked:

*"I think it is important and it has its place but I think that currently there is no one taking control about the initiation of it and the reasons for initiating it. There would be a lack of understanding about it in the primary care community" *(GP 13).

Reservations were also expressed on the introduction of new innovations:

*"Well its fairly negative. I mean its ...one of the problems we have within the health service generally is that new systems, new ways of dealing with patients are developed but no one really calculates or follows through the ramifications of implementing that. It may well be great for an individual patient but actually in the long term the care for that groups of patients has to be thought of as well" *(GP 4).

Attitudes to enteral feeding were linked to doctors' previous experiences, where those who had problems tended to be more negative than those who had none. Doctors who had patients in nursing homes found that they were addressing tube problems that the nursing staff could not deal with. One GP responded:

*"most of the nursing homes are able to do the initial interventions that are required to try and unblock tubes and basically we are called on when they cannot go any further and it needs to be looked at in day procedure or whatever" *(GP 10).

### Problem areas

Lack of experience coupled with no training was highlighted as a problem for some. As one GP remarked:

*"there has been a problem with the PEG tube and I have been asked to sort something out, I feel I don't really have much experience to do that" *(GP 18).

In relation to training, another respondent stated:

*"The training has been non existent. This is something that has just landed with us. The first time I knew absolutely nothing about it and the next time I knew next to nothing about it. It has just been handed to us without any training" (GP 8)*.

Issues relating to care of the tube were also highlighted as demonstrated in the following two comments:

*"The changing of the tube is a major problem" *(GP 16, 19)

*"The three things that I thought about was the problems with particularly the blockage of tubes, the budgets and the training" *(GP 10).

Some doctors felt that because they did not know enough about the treatment their knowledge of the problems that may arise was also lacking:

*"I don't know enough about them to know what difficulties there are because I have only had one patient and they had no problems" *(GP 20).

### Patient selection

With two exceptions, GPs had no involvement in the decision to place a feeding tube in their patient. As one GP observed *"no they generally come home from hospital with the tube in place" *(GP 18). Another commented *"We have had absolutely no role in it" (GP 4)*. It was observed that it would not be appropriate to be involved in the decision making process in every case. However, the unique perspective of the GP was exemplified by one respondent who commented:

*"I do know about the family dynamics and the situation and the capabilities of the patients and the families to manage that. I don't expect that the crux of the decision should depend on what I say, although it would be nice to be involved in the decision making process but we have this barrier between primary and secondary care so I don't expect that I will be involved in it" *(GP 4).

Another typical response portrayed decision-making in hospitals as "...a one way patient process". It was in this discussion that tensions between primary and secondary care were evident with metaphors relating to barriers such as, "forced upon" "arrived on your doorstep" were used. Although only one GP specifically mentioned the term *"barrier" *(GP 4) between primary and secondary care, others mentioned that primary care was a *"dumping ground"*, (GP 7)*"just landed on our door step" *(GP 9) or *"forced upon GPs" *(GP 17)

### Funding issues

The GPs in this study felt that there were issues surrounding funding of enteral feeding. The feeling that funding was not following the patient from secondary care into primary care was apparent as demonstrated in the following statements from respondents:

*"The additional costs in primary care is really off loading from secondary to primary care, primary care is getting very little funds with loads of funds going into secondary care" *(GP 12)

*"If the patients are being looked after in the community the funds that it would take to supervise and look after these people in hospitals are not being accessed. It follows the patient is the terminology but as you know quite often it does not happen" *(GP 15).

*"I would have a fair idea that quite a lot of GPs would say we are not going to just take this on, this is not going to become an additional thing that we have to look after without some consideration of funding" *(GP 18).

### Discharge information

There was a range of responses from GPs about the lack of information received about patients from *"it does not tend to be as good as it could be" *(GP 2) to *"inadequate with a capital I" *(GP 10). Doctors did not want a lot of information, but did want to know that the patient was being discharged and wanted the information at that time not *"weeks later" *(GP 9)

### Training needs with respect to enteral feeding

Almost every respondent mentioned that they did need some basic training in the area. One GP stated that his *"current knowledge of enteral feeding could be written on the back of a postage stamp and not a very big one" *(GP 13). The lack of training and knowledge highlighted some difficulties that GPs had with this treatment:

*"There would perhaps be a feeling among some of my colleagues that this is a thing that is coming from hospital and again as usual with many other things that are forced upon GPs with no support and no help and they say right there it is and get on with it and GPs say hang on a minute we don't know anything about this, we haven't been trained, we don't know the ins and outs of this therefore it's not our baby" *(GP 18).

*"I think that most GPs would value education in that area and I think it is something they would be attracted to because it is something when someone says they have a PEG tube and you sort of look blankly and think goodness what am I going to do about that" *(GP 2).

*"We need to know something about it for two reasons, one to be able to provide a service and secondly people expect us to know something about it and if we are sounding rather lacking in knowledge it doesn't do very much for the confidence level" *(GP 5)

Those doctors who felt that they did not wish to be trained, however, did want "(their) *district nurses trained*" (GP 11) or a *"refresher on the problem areas" *(GP 16) With respect to how the training should be delivered there were different views as to what would and would not work. Approximately half of the respondents felt that the most effective training would be delivered locally and, more specifically, within the practice. Most wanted it to be short. As one GP commented *"well to be honest with you if you want to score well it should only be half an hour" *(GP 13) Another stated *"Practice learning sessions is probably, I think, the best way"*.

The internet, handouts and a small booklet were mentioned, as was a simulation course. One doctor observed:

*"that something written out or a guideline written out and sent out around hundreds of GPs is probably not very effective and what happens is when you eventually need it you cannot find it" *(GP 23).

Others wanted basic information, the chance to see the tubes and equipment and to ask questions from the facilitator. Most did not care who the facilitator was, *"so long as they knew something about it" *(GP 19). GPs also felt that training is much more appropriate when they have a patient rather than having random training sessions:

*"You could be very much up to scratch now and because you have no practice with it in five years time you are back to square one" (GP 11)*.

## Discussion

Qualitative research is participant centred; the flexibility of the interview allows the participant to focus upon the issues of personal significance rather than predetermined topics. It has, therefore, the potential to generate novel insights and contributes to the development of the literature by highlighting issues that could be measured quantitatively. Qualitative research uses small sample sizes with the aim of this study being to understand individual GP views. As a consequence of the small sample size and the self selected participants we cannot establish laws of cause and effect or make claims about populations or trends. There may be a local effect as the GPs were from one geographical location. There may also be an element of selection bias; GPs with very negative experiences in the management of enteral feeding may have been more inclined to contact the research team.

This study does provide valuable information about the thoughts and experiences and the training needs of the GPs involved in the study. It also supports other studies about the topic [3] and work from other clinical areas such as palliative care where improved communication, lack of training and resources have also been identified as key issues [11-14].

While just under half felt that HETF was a positive treatment for patients, others had serious concerns about the management of patients in the primary care setting. The picture emerged of GPs who felt excluded from the decision to initiate enteral feeding in the community, that no-one had thought through the implications, and that they were unsupported in the community. They were not consulted about discharge in a situation where GPs, in particular, have a unique perspective on the family and background of the patient. Even when patients were in nursing homes GPs felt they had inadequate expertise as they were called in when the nursing staff were unable to sort out a problem. They believed that there are both resource and training issues to be addressed. But, perhaps the strongest message came from those GPs who felt that primary care was used as a *"dumping ground"*, that patients with HETF *"just landed on our door step" *or were *"forced upon GPs"*.

The anger expressed at the discharge of patients, and frustration with the poor or non-existent information received from secondary care was similar to findings in a study of diabetes care where the authors highlighted the need for better communication and cooperation between primary and secondary care and the need to develop initiatives to improve the primary-secondary care interface [11]. Scott and Wandsworth also identified poor communication rather than actual increases in workload as the biggest problem for GPs [7]. The issues identified here mirror the findings of other work where physicians have cited a lack of training, lack of time, lack of support staff with nutrition training and reimbursement concerns as barriers to their effective involvement in providing nutrition related information to patients [15-17].

Hospitals clinicians placing feeding tubes should consult GPs about the patients that they feel are suitable for feeding long term. GPs can provide the hospital clinicians with valuable information about the social circumstances and support, or lack of it, in the home environment. There are also difficulties with the lack of timely discharge information. By improving communication through these routes some of the problems that are being faced by GPs in primary care could be addressed.

Most GPs did not want to be experts in the area, as they were only ever going to have small numbers of patients. They did want some basic information delivered at practice level to allow them to manage both the difficulties in enteral feeding and problems that may arise with patients on an enteral feeding system. The education packages to support HETF should be targeted at those GPs managing HETF, timely, concise, appropriate to the patient and with clear and shared guidance for the patient, GP and specialist. Given the small patient numbers and the potential time lapse between patients, the education should be delivered each time a GP has a patient discharged.

In Northern Ireland community dietitians, who have other clinical commitments and/or health promotion duties, review patients. Patients are rarely reviewed by hospital outreach (some specialist units such as renal patients and cystic fibrosis may review their patients) and this may offer some reason as to the difficulties that GPs have reported in this study. Within the Eastern Health and Social Services Board (EHSSB) Area of Northern Ireland community dietetic services were reorganised in 1997 following an audit of HETF [18]. This allowed one full time community dietitian to develop the service and meet the recommendations, which suggested that patients should be reviewed at least every six months. Since then, because of increased numbers (approximately 250 adult patients from a population of 600,000), there are now two community dietitians providing this service to adult patients (EHSSB statistics, personal communication). In the UK different approaches to monitoring exist. In Leicestershire, a home enteral nutrition service was established in the early nineties to meet the needs of patients and their carers [19]. Similar dietetic-led services have been developed in other areas, for example, Avon and London. In Avon, service changes have been facilitated by changes in financial arrangements within the area [20], which again have facilitated staff to manage the patients and provide the necessary equipment for the process.

Pulling together the practical aspects of HETF, the emotional, social and educational issues associated with tube feeding rely heavily on trained practitioners in primary care. Larger caseloads will allow practitioners to gain experience along with formal continuing education and professional development [20].

## Conclusion

GPs should be included in the selection process and have adequate discharge information about their patients. Lack of training is a major issue for GPs and a simple focused training programme may improve their management of enteral feeding in the community. There was anger and frustration among GPs about the lack of resources, poor communication and a perception that primary care was used as a dumping ground.

## Competing interests

The author(s) declare that they have no competing interests.

## Authors' contributions

SMM carried out the interviews and the analysis of the data and drafted the manuscript. PF and SMcC carried out analysis of the interview transcripts. SMM, PF, MEW and DMacA conceived the study, participated in the design of the study and coordination and helped to draft the manuscript. All authors read and approved the final manuscript.

## Pre-publication history

The pre-publication history for this paper can be accessed here:


